# Pre-proenkephalin 1 is Downregulated Under Unloading and is Involved in Osteoblast Biology

**DOI:** 10.1007/s00223-024-01199-z

**Published:** 2024-03-20

**Authors:** Chiara Puri, Charlotte Dannenberg, Argia Ucci, Marco Ponzetti, Elisa Pucci, Luciana Silvestri, Patrick Lau, Petra Frings-Meuthen, Martina Heer, Nadia Rucci, Anna Teti, Antonio Maurizi

**Affiliations:** 1 Department of Biotechnological and Applied Clinical Sciences, University of L’Aquila, Via Vetoio - Coppito 2, 67100 L’Aquila, Italy; 2 German Aerospace Center (DLR), Institute of Aerospace Medicine, Cologne, Germany; 3 Institute of Nutritional and Food Sciences, University of Bonn, Bonn, Germany

**Keywords:** Pre-proenkephalin1 (Penk1), Mechanical unloading, Osteoblast differentiation

## Abstract

Pre-proenkephalin 1 (Penk1) is a pro-neuropeptide that belongs to the typical opioid peptide’s family, having analgesic properties. We previously found *Penk1* to be the most downregulated gene in a whole gene profiling analysis performed in osteoblasts subjected to microgravity as a model of mechanical unloading. In this work, *Penk1* downregulation was confirmed in the bones of two in vivo models of mechanical unloading: tail-suspended and botulinum toxin A (botox)-injected mice. Consistently, in the sera from healthy volunteers subjected to bed rest, we observed an inverse correlation between PENK1 and bed rest duration. These results prompted us to investigate a role for this factor in bone. *Penk1* was highly expressed in mouse bone, but its global deletion failed to impact bone metabolism in vivo. Indeed, *Penk1* knock out (*Penk1*^*−/−*^) mice did not show an overt bone phenotype compared to the WT littermates. Conversely, in vitro* Penk1* gene expression progressively increased during osteoblast differentiation and its transient silencing in mature osteoblasts by siRNAs upregulated the transcription of the *Sost1* gene encoding sclerostin, and decreased *Wnt3a* and *Col1a1* mRNAs, suggesting an altered osteoblast activity due to an impairment of the Wnt pathway*.* In line with this, osteoblasts treated with the *Penk1* encoded peptide, Met-enkephalin, showed an increase of *Osx* and *Col1a1* mRNAs and enhanced nodule mineralization. Interestingly, primary osteoblasts isolated from *Penk1*^*−/−*^ mice showed lower metabolic activity, ALP activity, and nodule mineralization, as well as a lower number of CFU-F compared to osteoblasts isolated from WT mice, suggesting that, unlike the transient inhibition, the chronic *Penk1* deletion affects both osteoblast differentiation and activity. Taken together, these results highlight a role for Penk1 in the regulation of the response of the bone to mechanical unloading, potentially acting on osteoblast differentiation and activity in a cell-autonomous manner.

**Supplementary Information:**

The online version contains supplementary material available at 10.1007/s00223-024-01199-z.

## Introduction

Recent research has shown that neuropeptides affect the functions of bone cells playing an essential role in the metabolism of the skeletal tissues [1, 2]. Pre-proenkephalin 1 (*Penk1*) is a gene encoding a pro-neuropeptide that is proteolytically processed to generate the following enkephalins: [Met]-enkephalin, [Met]-enkephalin extended sequence, and [Leu]-enkephalin, all belonging to the family of opioid peptides that are endogenous ligands for opioid receptors having potent analgesic properties [3]. In addition to their analgesic effects, enkephalins also regulate mood, stress, and addiction [4]. Dysfunction of the enkephalin system has been implicated in a range of psychiatric and neurological disorders, in chronic pain, cardiac and gastrointestinal functions, and immunity [5–10]. Coherently with the broad range of functions, their expression is not limited to the nervous and neuroendocrine systems. Indeed, *Penk1* mRNA is particularly abundant during development [11] and is expressed in many tissues, such as kidney, liver, lung, skeletal muscle, and heart [12, 13].

Our interest in *Penk1* started from a previous study of global gene profile analysis performed in mouse primary osteoblasts subjected to simulated microgravity by employing the NASA-developed Rotating Wall Vessel (RWV) apparatus as an in vitro model of mechanical unloading [14]. Among the differentially regulated genes observed in this study, some were not known to be associated with bone cell functions, thus representing new bone determinants. These included *Penk1*, which resulted to be the most downregulated osteoblast mechano-responding transcript in our experimental conditions [14]. Indeed, previous studies already demonstrated the expression of *Penk1* in bone and joints [15, 16]. Moreover, Rosen et al. identified *Penk1* mRNA in foetal rat calvaria-derived cells as well as in osteoblast-like cell lines [15, 17]. The same authors reported an inverse correlation between osteoblastic *Penk1* mRNA levels and cellular differentiation [18]. Later, Seitz et al. [19] found that, at variance with Rosen finding, *Penk1* was more expressed in differentiated osteoblasts, but its presence seemed to be dispensable for bone development, since bone histomorphometry did not reveal any bone defect in a mouse model deficient for *Penk1* [19, 20]. However, when *Penk1* was deleted in a spontaneous mouse model recapitulating the X-linked hypophosphatemic rickets disease (i.e. Hyp mouse), which harbours a deletion within the *Phex* gene encoding for an endopeptidase crucial for bone mineralization, they observed a reduction of the osteoid enrichment at 24 weeks of age [19]. Interestingly, Pérez-Castrillón and colleagues [21] found the expression of opioid receptors in the osteoblast-like MG63 cell line, suggesting the ability of these cells to respond to opioid stimulation.

Overall, the relationship between *Penk1* and bone metabolism is still an area of active research. In this context, we sought to investigate *Penk1* involvement in bone metabolism under physiological and mechanical unloading conditions. We confirmed, in in vivo mouse and human models, an inverse correlation between *Penk1* and unloading. However, we could not find any obvious phenotype in *Penk1*^*−/−*^ mice. In contrast, in ex vivo osteoblast primary cultures the lack of *Penk1* impaired Alkaline phosphatase (Alp) activity and nodule mineralization formation. Consistently, transient *Penk1* knock-down in differentiated osteoblasts by siRNAs treatment reduced the transcriptional expression of genes involved in bone formation, while treatment with the opioid receptor agonist Met-Enkephalin (Met-Enk) encoded by *Penk1* increased the transcriptional expression *Collagen 1α1* as well as nodule mineralization.

## Materials and Methods

### Materials

Dulbecco's modified Minimum Essential Medium (DMEM), penicillin, streptomycin, and trypsin were from Euroclone (Milan, Italy), Foetal Bovine Serum (FBS) was from GIBCO (Uxbridge, UK). Sterile plasticware was from Greiner bio-one (Kremsmünster, Austria) or Euroclone (Milan, Italy). TRIzol reagent (cat#15596018), primers and reagents for RT-PCR (cat#k1622) were from Invitrogen (Carlsbad, CA, USA). The qRT-PCR assays Luna Universal One‐Step RT‐qPCR Kit (#E3006) was from New England Biolab. Human (cat#CSB-EL017781HU) and mouse (cat#CSB-EL017781MO) PENK1 ELISA assays were from Cusabio (Houston, TX, USA); Carboxy Terminal collagen crosslinks (CTx, cat#AC-06F1), Procollagen type I N-terminal Propeptide (PINP1, cat#AC-33F1), and Tartrate-Resistant Acid Phosphatase (TRAcP, cat#SB-TR103) immunoenzymatic kits were from Immunodiagnostic Systems (The Boldons, UK). Met-Enkephalin (Met-Enk) was purchased by Sigma (cat# M6638). Anti-Collagen Iα1 antibody was from Immunological Sciences (cat#AB82138). All other reagents, including the histochemical Alp (cat #86C-1KT) and TRAcP (cat #386) kits, and Thiazolyl Blue Tetrazolium Bromide (cat# M2128), were of the purest grade from Sigma-Aldrich Co. (St. Louis, MO, USA). ON-TARGETplus Mouse Penk1-siRNA SMARTpool was purchased from Dharmacon (#L-050124-00-0005). Real-time array *mouse osteogenesis* (cat#pamm-026) was provided by SABiosciences (now part of QIAGEN).

### Animals

*Penk1*^*–/–*^ mice (background C57BL/6J) were purchased by the European Mouse Mutant Archive (EMMA), and their phenotype described in Konig et al*.* [20]. They are vital, with an overall normal lifespan and fertility. All procedures involving animals and their care were conducted in conformity with national and international laws and policies (European Economic Community Council Directive 86/609, OJ L 358, 1, December 12, 1987; Italian Legislative Decree 4.03.2014, n.26, Gazzetta Ufficiale della Repubblica Italiana no. 61, March 4, 2014) and animal procedures received approval by the Italian Ministry of Health authority (Approval N. 392/2020-PR). Mice were housed in the animal facility of the University of L’Aquila, Italy, at the following conditions: temperature: 20–24 °C, humidity: 60%, dark/light cycle: 12/12 h. They had access to food and water ad libitum and were fed with standard diet (Mucedola code: 4RF21).

### Hindlimb Suspension and In Vivo Treatment with Botulin Toxin A

Hindlimb suspension (HLS) was performed according to Sakata et al. [22] on 8-week-old C57BL/6J male mice and as described in Rucci et al. [23] while in vivo treatment with botulinum toxin A (Botox) was performed in 8-week-old C57BL/6J male mice according to Warner and colleagues [24] and as described in Rucci et al. [23].

### Head Down Tilt Bed Rest (HDBR) Experiment

A 14-day head-down tilt bed rest (HDBR) study, including 8 male subjects (mean age 26.3 ± 3.5 years, Body Weight [BW] 78.0 ± 4.3 kg) was conducted in the metabolic ward of the German Aerospace Center. Immobilization was accomplished with 6-degree HDBR, a valid ground-based model to simulate microgravity-induced bone loss. Study design and primary outcome are described more detailed in Frings-Meuthen and colleagues [25]. For the assessment of serum PENK1 levels in the present study, we took advantage of 3 unutilized study samples. Approval for the study was obtained from the Ethical Committee of the “Aerztekammer Nordrhein”, Düsseldorf, Germany, and was conducted in accordance with the latest version of the Declaration of Helsinki. The study is registered on http://www.clinicaltrials.gov with the unique trial number: NCT01183299; registration date: August 13, 2010.

### Osteoblast Primary Cultures

Primary mouse osteoblast cultures were performed according to [26] and cells were cultured in DMEM plus 10% FBS. Briefly, calvariae from 7-day-old CD1, C57BL/6J WT, and *Penk1*^*−/−*^ mice (both genders) were explanted, cleaned free of soft tissues, and digested three times with 1 mg/mL *Clostridium histolyticum* type IV collagenase and 0.25% trypsin, for 15, 30, and 45 min at 37 °C, with gentle agitation. Supernatant from the first digestion was discarded, while those of the second and third digestion were centrifuged at 300 g for 7 min and cells were resuspended and cultured in DMEM plus 10% FBS. At confluence, cells were trypsinized and plated according to the experimental protocol.

For siRNA transfection, mouse primary osteoblasts (1 × 10^6^ cells) were nucleofected with 100 nM of *Penk1*-specific or scrambled (SCR)-siRNA (Dharmacon) using the Amaxa Mouse Neuron Nucleofector kit (Lonza, Basel, Switzerland; Cat. #VPG-1001), after 7 days of culture. After 48 h from nucleofection, osteoblasts were employed for the experiments. Alp activity was evaluated cytochemically according to the manufacturer’s instructions. For mineralization assay, DMEM plus 10% FBS was supplemented with 10 mM β-glycerophosphate and 50 µg/mL ascorbate (osteogenic medium). Mouse primary osteoblasts were cultured for 3 weeks before evaluation of mineralization nodules by von Kossa staining. Metabolic activity was assessed by the 3-(4,5-dimethylthiazol-2-yl)-2,5-diphenyltetrazolium (MTT) bromide reduction assay, according to the manufacturer’s instructions.

Alp-positive colony forming unit (CFU) was performed according to Capulli et al. [26] and at the end of the experiment number and area of Alp-positive colonies were analysed to estimate the amount of osteoblast progenitors in the bone marrow, using the Fiji® software.

For the treatment with the opioid receptor agonist, [Met]-Enkephalin (Met-Enk), WT mouse primary osteoblasts were incubated with vehicle or 1 µM Met-Enk for 48 h using DMEM without FBS supplementation.

### Osteoclasts Primary Culture

Bone marrow flushed out from the bone cavity of the long bones of 7-day-old mice (CD1 background) was diluted 1:1 in Hank’s balanced salt solution, layered over Histopaque 1077 solution, and centrifuged at 400 g for 30 min. Cells were washed twice with Hank’s solution, resuspended in DMEM, and plated in culture dishes for 12 min. Then, non-adherent cells were recovered and plated in 96-well plate at density of 300.000 cells/well and maintained for 7 days in medium supplemented with 50 ng/mL rhMCSF and rhRANKL (120 ng/mL). At the end of the culture, conditioned medium was recovered and the level of Penk1 assessed by ELISA.

### Comparative Real-Time RT-PCR

Total RNA was extracted Using the TRIzol® method. One microgram of RNA was reverse transcribed into cDNA using Revert Aid First Strand cDNA Synthesis Kit (Invitrogen) and the equivalent of 0.1 μg was processed using the Luna Universal One‐Step RT‐qPCR Kit (New England Biolab). Results, expressed as fold increase *vs* control, were normalized with the housekeeping gene glycerol-3-phosphate dehydrogenase (*Gapdh*). Primer sequences are reported in Supplementary Table 1.

### MicroCT Analysis

Images of femurs previously fixed in 4% buffered paraformaldehyde were acquired using the SkyScan 1174 (Bruker, Billerica, MA, USA) with a resolution of 6.7 μm (X-ray voltage 50 kV). Image reconstruction was carried out employing a modified Feldkamp algorithm using the Skyscan Nrecon software. Three-dimensional (3D) and two-dimensional (2D) morphometric parameters were calculated for the trabecular bone, 100 slides (6.7 μm thick) from the growth plate [27]. 3D parameters were based on analysis of a Marching Cubes-type model with a rendered surface [28, 29]. Calculation of all 2D areas and perimeters was based on the Pratt algorithm [30]. Bone structural variables and nomenclature were those suggested by Bouxsein et al. [31]. Cortical bone thickness was analysed 450 slides below the growth plate on 54 slides as described [32].

### Bone Histomorphometry

Tibiae harvested from WT and *Penk1*^*−/−*^ mice were fixed in 4% buffered paraformaldehyde, dehydrated in ascending alcohol series, and processed for methyl–methacrylate embedding without decalcification. Histomorphometric measurements were carried out on 5-μm-thick sections using the image analysis software NIH ImageJ (RRID:SCR_003070 version 1.50i.) and with the suggested nomenclature [33]. Osteoclast number/bone surface (Oc.N/BS) and osteoclast surface/bone surface (Oc.S/BS%) were evaluated after histochemical staining for TRAcP activity, while osteoblast number/bone surface (Ob.N/BS) and osteoblast surface/bone surface (Ob.S/BS%) were evaluated in sections stained with toluidine blue. Dynamic assessment of the mineral apposition rate (MAR) was calculated after double injection of calcein, 10 and 3 days before euthanasia. Bone formation rate (BFR) was calculated according to the following formula: MAR × MS/BA, where MS = mineralized surface and BA = bone area, as suggested by Dempster and colleagues [33].

### Bone Turnover Biomarkers

Markers of bone turnover, including CTx, TRAcP 5b isoform, and PINP1 were evaluated in sera collected from WT and *Penk1*^*−/−*^ mice by ELISA according to the manufacturer’s instructions. ELISA kits information is reported in the Materials section.

### Statistics

Results are expressed as the mean ± SD of at least 3 independent experiments, 3 human subjects, and at least 4 mice/group. Statistical analyses were performed by the Student's *t* test or linear regression test according to the type of data, using the Prism Graph pad software (v8). Pearson’s correlation analysis was performed using the Prism Graph pad software (v8). A *p* value < 0.05 was conventionally considered statistically significant.

## Results

### Mechanical Unloading Induces *Penk1* Downregulation in Mouse and Human

**Fig. 1:**
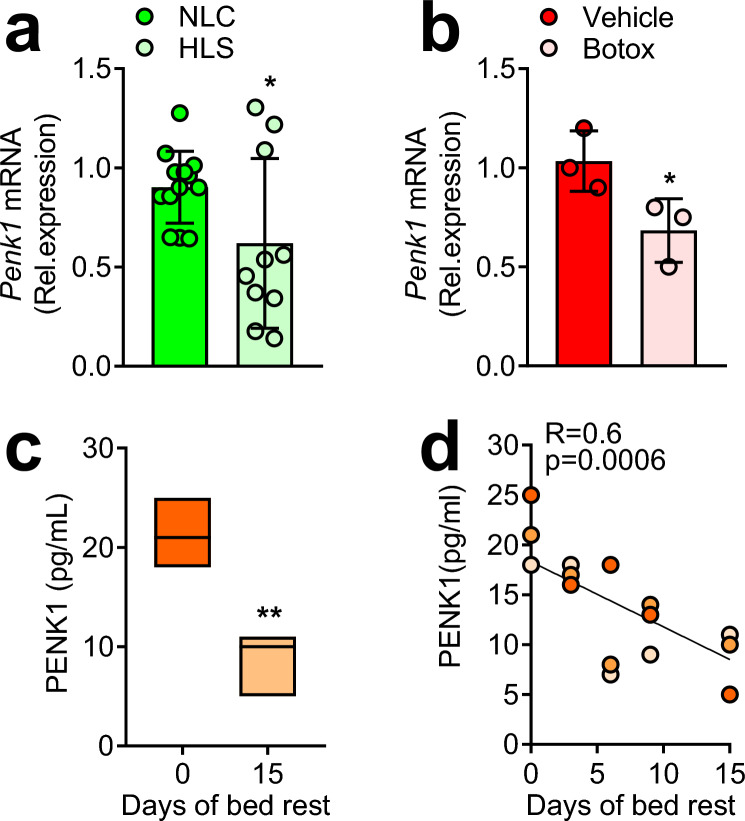
Penk1 modulation in mouse and human models of unloading. **a** Eight-week-old male mice were maintained in normal loading condition (NLC) or were subjected to hindlimb suspension (HLS) for 21 days. Transcriptional expression of *Penk1* in femurs by comparative real-time RT-PCR. **b** Eight-week-old mice were injected with saline solution (vehicle) or with Botulinum toxin A (Botox, 20 µL of 2.0 unit/100 g), into the right quadriceps and the posterior compartment of the right calf (targeting gastrocnemius, plantaris, and soleus) of the left or the right hindlimbs, respectively. Transcriptional expression of *Penk1* in femurs by comparative real-time RT-PCR. **c** Serum PENK1 was assessed by ELISA in the sera of human healthy volunteers at time 0 and after 14 days of bed rest. **d** Linear regression test showing an inverse correlation between serum PENK1 levels of human healthy volunteers subjected to bed rest and the duration of unloading. In **a**, **b** results are the mean ± SD of 3–10 mice/group. *p ≤ 0.05 (Student’s *t* test). Results in **c** are the mean ± SD of 3 human subjects per group. **p = 0.01 (Student’s *t* test)

Our interest in *Penk1* started from a previous work showing, by a whole genome microarray assay, that it was the most downregulated gene in osteoblasts subjected to microgravity as a model of in vitro mechanical unloading [14], suggesting a possible involvement of *Penk1* in the osteoblast mechano-response. These results prompted us to evaluate *Penk1* expression in two mouse models of disuse osteoporosis due to mechanical unloading: the tail-suspended mice and mice injected with botox in the right quadriceps and the posterior compartment of the right calf. Interestingly, we observed a significant reduction of *Penk1* mRNA expression in the femurs of mice subjected to hindlimb suspension (HLS) compared to mice maintained in normal loading condition (NLC, Fig. 1a). Consistently, a decrease of *Penk1* mRNA was also observed in the botox-injected hindlimbs, *vs* the contralateral limbs injected with vehicle (Fig. 1b). Penk1 concentration was not measurable in the mouse serum with the kit employed for the study. However, we had the chance to evaluate PENK1 expression in human sera from 3 healthy volunteers subjected to bed rest for 14 days, a well-known mechanical unloading model. Our analysis showed a significant reduction of PENK1 serum level after 14 days of bed rest (Fig. 1c) along with an inverse correlation between PENK1 serum levels and duration of bed rest (Fig. 1d). Taken together, these results indicate that mechanical unloading causes a reduction of Penk1 expression systemically, as observed in humans, or locally in bone, as observed in mice, and this could mediate the response of the skeleton to this condition.

### Lack of an Overt Bone Phenotype in *Penk1* KO Mice

**Fig. 2:**
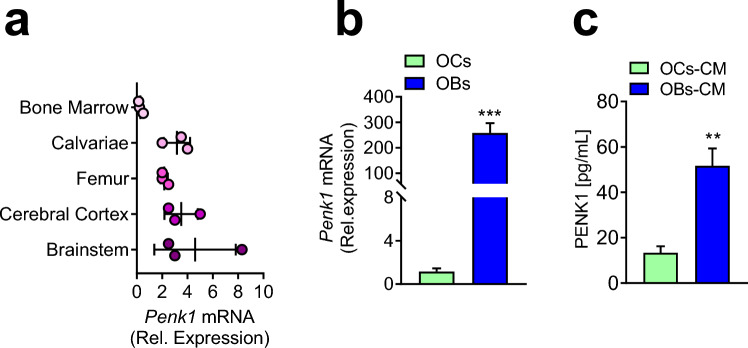
*Penk1* expression. **a** Transcriptional expression of *Penk1* evaluated by real-time RT-PCR in the indicated organs and tissues collected from 3-month-old WT male mice. **b** Transcriptional expression of *Penk1* in mouse primary osteoclasts (OCs) and osteoblasts (OBs). **c** Penk1 protein levels in conditioned medium harvested from mouse osteoclast (OCs-CM) and osteoblast (OBs-CM) primary cultures. Gene expression was normalized using mouse *Gapdh*. Data are the mean ± SD of at least 3 mice or 3 cell preparations per group. **p = 0.0012; ***p ≤ 0.001. (Student’s *t*)

**Fig. 3:**
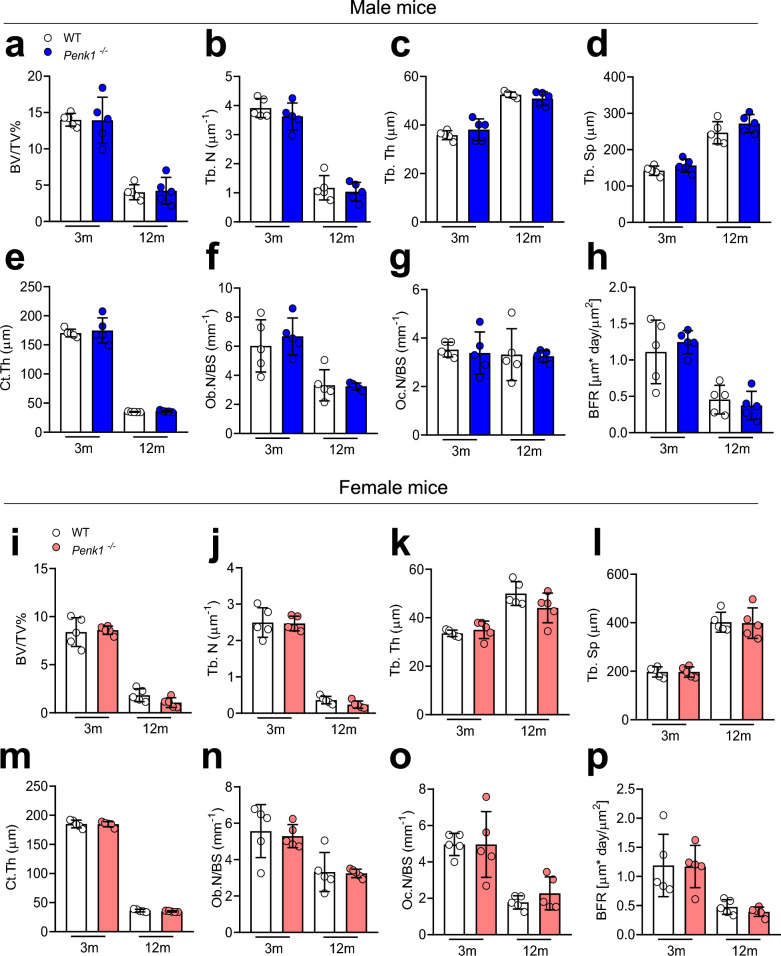
Bone phenotype of *Penk1* globally deleted mouse model. MicroCT analysis of distal femurs isolated from 3- and 12-month-old WT and *Penk1*^*−/−*^ **a**–**d** male and **i**–**l** female mice to evaluate trabecular and cortical bone structural variables. After μCT scanning, bones were embedded in plastic, sectioned, stained with Toluidine blue and evaluated histomorphometrically for **f**, **n** Osteoblast Number over Bone Surface (Ob.N/BS) and for TRAcP enzymatic activity to evaluate **g**, **o** the Osteoclast Number over Bone Surface (Oc.N/BS). Double in vivo calcein labelling was performed to evaluate **h**, **p** the Bone Formation Rate over Bone Surface BFR/BS. Data are the mean ± SD of 5 mice per group. (Student’s *t*)

Based on the effect of the microgravity on *Penk1* expression, we aimed at characterizing the role of *Penk1* in vivo. Interestingly, besides the brainstem and the cerebellar cortex, already expected to express *Penk1* mRNA, we found a comparable transcriptional expression of this gene in mouse femurs and calvariae cleaned from bone marrow, while its expression in the flush-out bone marrow cells was very low (Fig. 2a). Further examination revealed that, among the bone cells evaluated, the osteoblasts expressed significantly higher transcriptional and protein levels of Penk1 compared to osteoclasts (Fig. 2b, c). However, in line with Seitz et al. [19], μCT analysis on tibiae of *Penk1*^*−/−*^ mice did not show any difference in bone volume over tissue volume between the two genotypes at 3 and 12 months of age in both male and female mice (Fig. 3a, i). Consistently, evaluation of trabecular parameters showed that the trabecular number (Tb.N, Fig. 3b, j), thickness (Tb.Th, Fig. 3c, k), and separation (Tb.Sp, Fig. 3d, l), as well as the cortical bone thickness (Ct.th, Fig. 3e, m), were not influenced by the lack of *Penk1*. As expected, histomorphometry revealed that osteoclast number/bone surface (Oc.N/BS), osteoclast surface/bone surface (Oc.S/BS %), osteoblast number/bone surface (Ob.N/BS), and osteoblast surface/bone surface (Ob.S/BS%) were similar between the two genotypes (Fig. 3f, g, n–o, and Supplementary Tables 2, 3), along with the bone dynamic parameters Mineral Apposition Rate (MAR) and Bone Formation Rate (BFR) (Fig. 3h, p, and Supplementary Tables 2, 3), both in males and females. Finally, no differences were observed in the serum bone remodelling biomarkers TRAcP, CTx, and PINP1 (Table 1). Therefore, despite the results we found in microgravity, these data confirmed that the systemic ablation of *Penk1* in vivo does not affect bone metabolism in normal mechanical conditions. These results can be partially explained by the combined effect of residual enkephalins and other neuropeptides on bone cells that could potentially counterbalance the effect of the absence of Penk1 in vivo.

**Table 1:** Serum bone remodelling biomarkers in WT and *Penk1*^*−/−*^ mice

3-Month-old	Males			Females		
	WT	*Penk1*^*−/−*^	*p* value	WT	*Penk1*^*−/−*^	*p* value
TRAcP (U/L)	7.17	8.70	0.15	7.96	7.97	0.99
CTx (ng/mL)	12.22	15.12	0.50	27.55	33.62	0.18
PINP (ng/mL)	35.31	67.36	0.13	46.6	38.80	0.58
12-Month-old						
TRAcP (U/L)	2.25	2.31	0.94	4.07	3.84	0.82
CTx (ng/mL)	19.74	17.04	0.43	22.17	21.32	0.76
PINP (ng/mL)	47.29	53.07	0.81	41.90	61.66	0.29

### Penk1 Regulates Osteoblast Differentiation and Activity

**Fig. 4:**
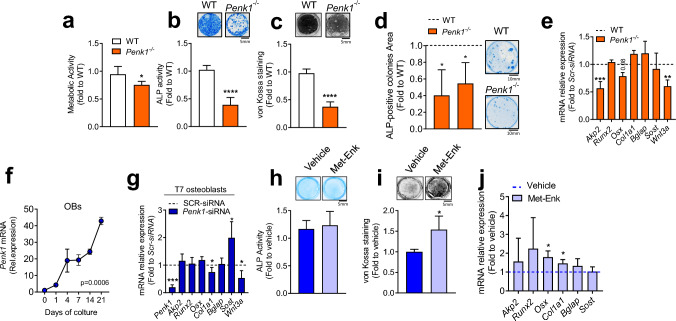
Effect of Penk1 in osteoblast biology. Primary osteoblasts were isolated from the calvariae of 7-day-old WT and *Penk1*^*−/−*^ mice and evaluated for **a** cell metabolic activity assessed by the 3-(4,5-dimethylthiazol-2-yl)-2,5-diphenyltetrazolium (MTT) bromide reduction assay, **b** ALP activity by cytochemical assay and quantified by densitometric analysis (graph), and **c** von Kossa staining of mineralization nodules after 3 weeks of culture in osteogenic medium. **d** Osteogenic colony forming unit assay performed in bone marrow stromal cells flushed out from femurs of 7-day-old WT and *Penk1*^*−/−*^ mice. **e** Osteoblast differentiation markers analysed by real-time RT-PCR. **f** Time-course of *Penk1* mRNA expression in WT primary osteoblasts cultured in standard medium supplemented with 10 mM β-glycerophosphate and 50 µg/mL ascorbic acid (osteogenic medium). Mouse primary osteoblasts were treated with scrambled (SCR) siRNA or with siRNAs specific for *Penk1* (*Penk1*-siRNA). **g** After 48 h the RNA was extracted, retro-transcribed in cDNA, and subjected to real-time RT-PCR for the indicated genes. Mouse primary osteoblasts were treated with vehicle or 1 µM Met-Enkephalin (Met-Enk) for 48 h. **h** ALP activity evaluated by histochemical assay and quantified by densitometric analysis (graph) and **i** von Kossa staining to evaluate nodule mineralization in osteoblasts treated with vehicle or 1 µM Met-Enk for 2 weeks in the presence of osteogenic medium. **j** Transcriptional expression of the genes indicated in the abscissa, evaluated by real-time RT-PCR, in Met-Enk-treated osteoblasts. Scale bars are indicated in the figures. Gene expression was normalized using mouse *Gapdh.* Data are the mean ± SD of at least 3 mice or 3 cell preparations per group. *p ≤ 0.05; **p = 0.0012; ***p ≤ 0.001; ****p ≤ 0.0001 (Student’s *t* test or in **e** RM-ANOVA)

To remove any confounding effect related to compensatory mechanism acting in vivo, we next investigated a potential role for *Penk1* in osteoblasts. Despite the absence of an overt bone phenotype in vivo, primary osteoblasts isolated from *Penk1*^−/−^ mouse calvariae showed an impairment of their metabolic and ALP activities along with a lower nodule mineralization ability, compared to cells isolated from WT mice (Fig. 4a–c). In a CFU-Fibroblastic assay, using bone marrow cells isolated from tibias, we observed a lower number and area ALP-positive colonies in *Penk1*^*−/−*^ vs WT mice (Fig. 4d), suggesting a possible cell-autonomous positive effect of *Penk1* in the commitment of progenitor cells to the osteoblast lineage. In line with this, gene expression analysis showed a lower *Akp2*, encoding Alkaline phosphatase (Alp) and Wnt3a expression, and a trend of reduction in *Osterix* (*Osx*) mRNA level in *Penk1*^*−/−*^ vs WT primary osteoblasts (Fig. 4e).

In agreement with these observations, we found that *Penk1* transcriptional expression increased during osteoblast differentiation in mouse primary osteoblasts cultured in osteogenic medium (Fig. 4f). This positive relationship between *Penk1* and osteoblast differentiation was also confirmed by correlation analysis. Indeed, in addition to the positive correlation between the duration of the culture and *Penk1* mRNA level (Supplementary Fig. 1a), this latter also correlated positively with the mRNA expression levels of *Osx*, *Runx2*, *Akp2*, and *Bone gamma-carboxyglutamic acid-containing protein (Bglap)* encoding Osteocalcin, during osteoblast differentiation (Supplementary Fig. 1b–d, f). A trend of positive correlation was likewise found between *Penk1* and *Col1a1* (Supplementary Fig. 1e).

Interestingly, *Penk1* mRNA downregulation in wild type osteoblasts by treatment with specific *Penk1* siRNAs increased *Sclerostin* (*Sost*) while reducing *Wnt3a* and *Col1α1* mRNAs, compared to cells treated with scrambled siRNA (SCR-siRNA, Fig. 4g). As expected, *Sost* expression in control SCR-treated osteoblasts was low and increased by *Penk1*-siRNA treatment (Supplementary Fig. 1). Consistently, *mouse osteogenesis* real-time RT-PCR array confirmed that *Sost* was the most upregulated gene upon *Penk1*-siRNA treatment along with a reduced expression of *Bone Morphogenic Protein* (*Bmp*) 1 and 3 (Supplementary Table 2). Interestingly, we also found a positive correlation between *Sost* and *Penk1* expression during osteoblast differentiation (Supplementary Fig. 2 g). In contrast, osteoblast differentiation markers, including *Akp2*, *Runx2*, *Osterix* (*Osx*), and *Bone gamma-carboxyglutamic acid-containing protein (Bglap)* encoding Osteocalcin, were unremarkable (Fig. 4g), indicating a possible role of Penk1 in Wnt-mediated osteoblast anabolic activity rather than in osteoblast differentiation when downregulated in mature cells. In line with this observation, treatment of primary osteoblasts with [Met]-Enkephalin (Met-Enk), an agonist of the *δ*- and *μ*-opioid receptors derived from the proteolytic cleavage of Penk1, did not affect Alp activity (Fig. 4h), while it increased nodule mineralization compared to vehicle-treated cells (Fig. 4i). Consistently, transcriptional analysis showed a significant increase of *Col1a1* and *Osx* in Met-Enk-treated cells (Fig. 4l).

Altogether these data demonstrated that Penk1 affects both osteoblast function and differentiation in a cell-autonomous manner, and that the transient deletion of *Penk1* in differentiated osteoblasts specifically impairs the osteoanabolic activity while its chronic deletion affects both differentiation and activity.

## Discussion

Neuropeptides are involved in the regulation of bone metabolism, influencing the activity of bone cells, including osteoblasts, osteoclasts, and osteocytes [1, 2, 34, 35]. Nevertheless, the role in bone of opioids, a specific class of neuropeptides, is still partially unclear and the complexity of their signalling pathway further complicates the scenario. Indeed, opioid receptors are known to be expressed in bone cells and opioid-derived peptides have been shown to have direct effects on bone metabolism [1, 17, 35, 36]. When opioids bind to their receptors on bone cells, they can activate several signalling pathways that influence bone cell functions. As an example, the use of naltrexone and naloxone, two opioid growth factor receptor (OGFR) antagonists, stimulate bone formation in vitro and vivo [37, 38]. Similar results were obtained in mice in which the expression of the opioid peptide dynorphin or its κ-opioid receptor were abolished [39]. In contrast, D’Ângelo et al. [40] demonstrated that the blockade of the κ-opioid receptor increases osteoclast differentiation and bone resorption in rats. Seitz et al. [19] observed a significant reduction of the osteoid formation in Phex-deficient Hyp mice in which *Penk1* expression was abolished in vivo, suggesting that Penk1 is involved in the accumulation of non-mineralized bone matrix. A recent study showed that Met-Enk blocks osteocyte apoptosis by regulating the nuclear translocation of NFATc1 in the presence of compressive forces [34]. Finally, different studies have shown that the chronic use of opioids can lead to decreased bone density and increased risk of fractures [41, 42].

It is therefore within this intricate context that our study is inserted, with the scope of understanding the role of Penk1 in bone homeostasis under physiological conditions and in response to mechanical unloading. In line with our previous study, in which *Penk1* mRNA was downregulated in unloaded osteoblasts, in the present work we found that mechanical unloading leads to a reduction of *Penk1* expression both in mouse’s and human’s models of disuse osteoporosis. Moreover, we demonstrated an inverse correlation between Penk1 serum levels and the time of unloading, suggesting that Penk1 is highly responsive to this condition. These results not only suggest a possible role for Penk1 in the response of the skeleton to unloading conditions but they also candidate Penk1 as a possible marker of unloading-induced bone loss. Further study, including a larger cohort of human subjects, will be required to shed light on the underlining mechanisms. Indeed, the falls of Penk1 level could be also involved in the onset of chronic bone pain afflicting people exposed to skeletal unlading condition, such as astronauts and bedridden people [43, 44].

Despite the data obtained in the microgravity experiments, no impact on bone phenotype was observed in vivo in *Penk1*^−/−^ mice, compared to wild type littermates. These findings are in line with the results reported by Seinz et al. [19] on the lack of an overt bone phenotype in *Penk1*^−/−^ mice. They could be in part explained by the fact that residual-circulating enkephalins are detectable in the sera of *Penk1*^−/−^ mice, as demonstrated by [19]. A further explanation could be that the δ, κ, and µ opioid receptors expressed by bone not only bind Penk1-derived enkephalins, but also other neuropeptides, such as β-endorphins and dynorphins, known to impact the bone metabolism [17, 39, 40, 45]. Therefore, the combined effect of residual enkephalins and other neuropeptides on bone cells could potentially counterbalance the effect of the absence of Penk1 in vivo. This conclusion was further supported by the presence of impaired differentiation and activity in vitro in osteoblasts isolated from *Penk1*^−/−^ mice, suggesting the presence of a cell-autonomous effect in these cells. Moreover, we demonstrated that *Penk1* may be required for the commitment of progenitor cells to the osteoblast lineage.

To further recognize the involvement of Penk1 in bone metabolism, we investigated its role in osteoblast differentiation and activity. We found that Penk1 is highly expressed in bone and in mature osteoblasts. In this regard, conflicting data are reported in the literature. Indeed, Rosen et al. [18] demonstrated an inverse correlation between *Penk1* mRNA expression and osteoblast differentiation, while Seitz and colleagues [19] found a higher expression of *Penk1* in differentiated cells. In our hands, *Penk1* is expressed by the bone tissue, increasing during osteoblast differentiation, and its suppression impacts on osteoblast differentiation and activity. Interestingly, in our experimental condition, we found that the transient deletion of *Penk1* in differentiated osteoblasts, which express higher level of *Penk1* compared to the non-fully differentiated cells, impairs specifically osteoanabolic activity but not differentiation, while its chronic germline deletion affects both differentiation and activity. These findings are in line with a possible dual role of osteoblastic Penk1 in embryos and adults, as postulated by Rosen and Bar-Shavit [46]. To better understand the effect of *Penk1* knocking-down by siRNAs, we chose a time point during osteoblast differentiation (7 days) in which the *Penk1* expression was remarkable and stable. Nevertheless, at variance with the germline deletion, the timing of the knock-down experiments could hide possible effects of *Penk1* transient deletion on the early differentiation genes, such as *Osx*, *Runx2*, and *Akp2*. This limitation was further confirmed by the fact that *Penk1* mRNA was positively correlated with *Osx*, *Runx2*, *Akp2*, and *Bglap* mRNA levels during osteoblast differentiation. Moreover, in contrast with the observations of Nikhil et al. [38], we found that treatment of mature osteoblasts with the δ- and μ-opioid receptor agonist, [Met]-Enkephalin, increases nodule mineralization in vitro, also impacting the osteoblast differentiation, as suggested by the increased *Osx* expression. To reconcile these results, we can speculate that probably Met-Enk increases nodule mineralization indirectly, by enhancing type I collagen expression and bone matrix deposition, without acting directly on mineral metabolism, as shown by our gene expression data and in part suggested by Seinz et al. [19].

Mechanistically, we demonstrated that the Wnt pathway could be a potential candidate through which the Penk1 signalling acts on differentiated osteoblast activity. In fact, we showed that transient *Penk1* knock-down in differentiated osteoblasts increased the expression of the sclerostin encoding gene, *Sost1,* along with a parallel downregulation of *Wnt3a* and *Col1α1*. Of note, reduced level of *Wnt3a* mRNA was detected in primary osteoblasts isolated from Penk1^−/−^ mice, further confirming a possible involvement of the Wnt pathway in this context. As expected, *Sost* was expressed by osteoblasts at low level [47] and the treatment with *Penk1*-specific siRNA increased its expression. The relationship between *Penk1* and *Sost* was also confirmed by correlation analysis performed during osteoblast differentiation. Of note, in line with a potential role of the Wnt pathway, we also found a reduction of *Bmp2* gene expression in *Penk1* knock-down osteoblasts. Indeed, Wnt signalling pathway has been demonstrated to be an upstream activator of BMP2 expression in osteoblasts [48] and BMP2 in turn is known to activate the osteoblast Wnt pathway through Wnt3a [49].

In conclusion, our work adds another piece of information to the intricated network of interactions between neuropeptides and skeletal tissues, confirming a role of *Penk1* in osteoblast differentiation and activity and paving the way to future studies for dissecting the mechanisms underlining the involvement of Penk1 in unloading-induced bone loss.

### Supplementary Information

Below is the link to the electronic supplementary material.

Supplementary file1 (DOCX 253 kb)
