# Supplementary material for: Pre-proenkephalin 1 is Downregulated Under Unloading and is Involved in Osteoblast Biology
Source: Calcif Tissue Int. 2024 Mar 20;114(5):524–34. doi: 10.1007/s00223-024-01199-z (PMC11061007; doi:10.1007/s00223-024-01199-z)
Supplement: Supplementary file 1 — Supplementary file1 (DOCX 253 kb) [file 223_2024_1199_MOESM1_ESM.docx]

**Supplementary Figure 1.** Semiquantitative RT-PCR of the indicated genes performed on RNA isolated from wild-type primary mouse osteoblasts treated with 100nM of scrambled (SCR-) siRNA or *Penk1* specific siRNA (*Penk1*-siRNA). PCR products were resolved on 2% agarose gel. Pictures are representative of 3 experiments.

**
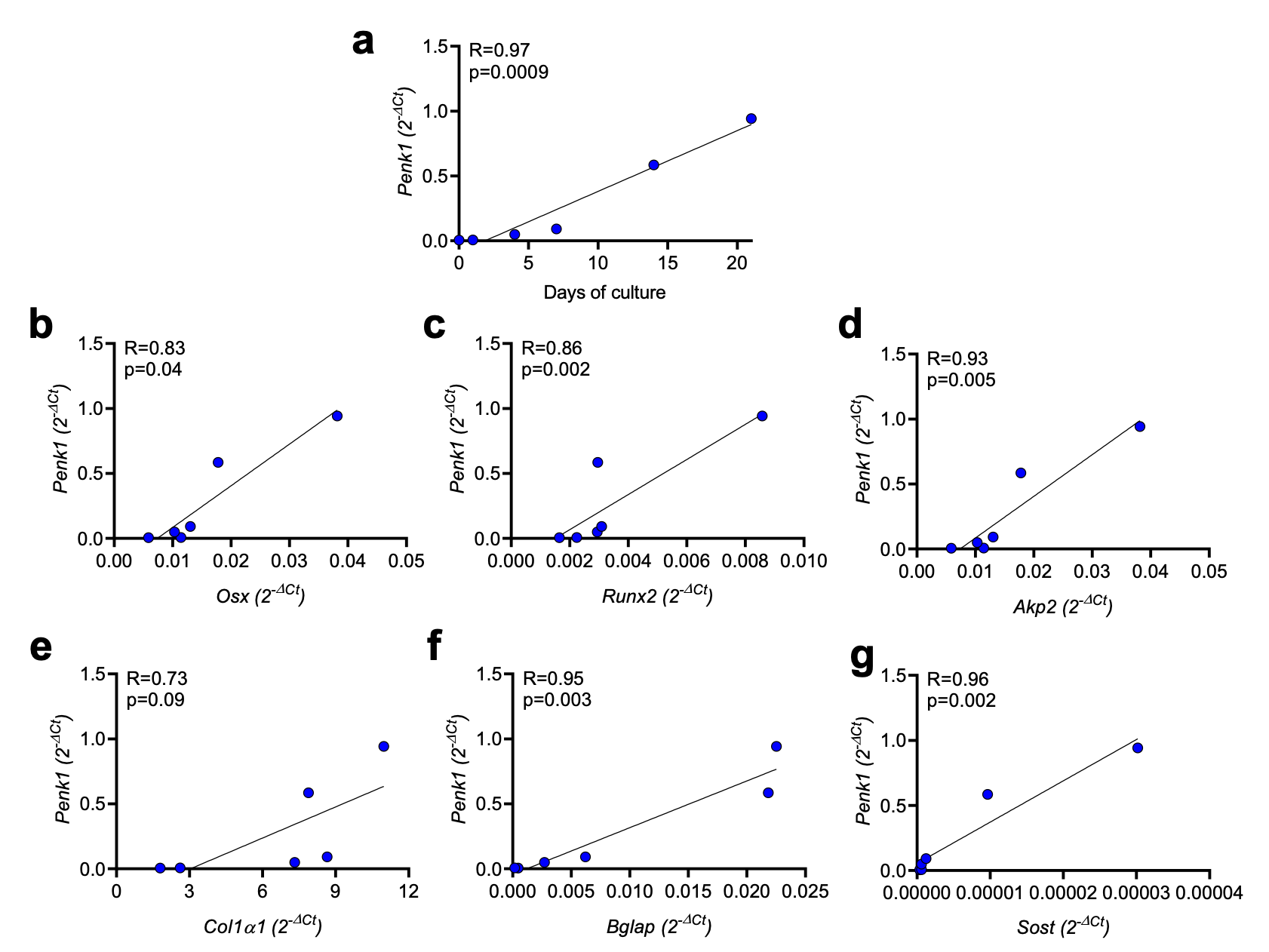
**

**Supplementary Figure 2. Correlation analysis.** Wild-type primary mouse osteoblasts were cultured in standard medium supplemented with 10 mM β-glycerophosphate and 50 µg/mL ascorbic acid (osteogenic medium). RNA was isolated at T0, T1, T4, T7, T14 and T21 culture days and the indicated genes were evaluated by real time RT-PCR. Correlation analysis was performed between *Penk1* and the indicated osteoblast differentiation markers. Genes expression is reported as 2^-ΔCt^. Pearson’s correlation analysis. R and p values are shown in the graph.

**Supplementary table 1.** Real time primers sequences.

| Gene | Forward primer | Reverse primer |
| --- | --- | --- |
| *Gapdh* | 5′-TGTGAGGGAGATGCTCAGT-3′ | 5′-TGTTCCTACCCCCAATGTG-3′ |
| *Penk1* | 5′-GACAGCAGCAAACAGGATGA-3′ | 5′-GTTGTCTCCCGTTCCCAGTA-3′ |
| *Akp2* | 5′-CCAGCAGGTTTCTCTCTTGG-3′ | 5′-CTGGGAGTCTCATCCTGAGC-3′ |
| *Runx2* | 5′-CCCAGCCACCTTTACCTACA-3′ | 5′-TATGGAGTGCTGCTGGTCTG-3′ |
| *Osx* | 5′-TGCTTCCCAATCCTATTTGC-3′ | 5′-AGAATCCCTTTCCCTCTCCA-3′ |
| *Col1α1* | 5′-CACCCTCAAGAGCCTGAGTC-3′ | 5′-GTTCGGGCTGATGTACCAGT-3′ |
| *Bglap* | 5′-TTCTGCTCACTCTGCTGACC-3′ | 5′-GGGACTGAGGCTCCAAGGT-3′ |
| *Sost* | 5′-ACAAGGATGGGAGGTGACTG-3′ | 5′-ACCCCGTGTAGACTGGTGAG-3′ |
| *RankL* | 5′-AGCCGAGACTACGGCAAGTA-3′ | 5′-CCACAATGTGTTGCAGTTCC-3′ |
| *Opg* | 5′-AGTCCGTGAAGCAGGAGTG-3′ | 5′-CCATCTGGACATTTTTTGCAAA-3′ |
| *Il-1β* | 5′-GCCCATCCTCTGTGACTCAT-3′ | 5′-AGGCCACAGGTATTTTGTCG-3′ |
| *Il-6* | 5′-GTTCTCTGGGAAATCGTGGA-3′ | 5′-GGAAATTGGGGTAGGAAGGA-3′ |
| *Wnt3a* | 5′-TACCCGATCTGGTGGTCCTT-3′ | 5′-GGGCATGATCTCCACGTAGT-3′ |

**Supplementary Table 2.** Selection of the most significantly regulated genes expressed as the ratio between osteoblasts treated with *Penk1*-specific siRNA (*siPenk1*) and osteoblasts treated with scrambled siRNA (siSCR).

| **Gene Symbol** | **Gene Name** | **siPenk1/siSCR (fold change)** |
| --- | --- | --- |
|  | **Up-regulated genes** |  |
| *Sost* | Sclerostin | 33.17 |
| *Enam* | Enamelin | 6.28 |
| *Tfip11* | Tuftelin interacting protein 11 | 5.66 |
| *Itga2* | Integrin alpha-2 | 4.60 |
| *Nfkb1* | Nuclear factor kappa-b, subunit 1 | 2.74 |
| *Ambn* | Ameloblastin enamelmatrix protein | 2.71 |
| *Igf1r* | Insulin-line growth factor 1 receptor | 2.13 |
| *Vegfb* | Vascular endothelial growth factor beta | 2.03 |
|  | **Down-regulated genes** |  |
| *Col10a1* | Collagen 10a1 | 0.04 |
| *Egf* | Epidermal growth factor | 0.05 |
| *Itga3* | Integrin alpha-3 | 0.16 |
| *Bmp2* | Bone morphogenetic protein 2 | 0.16 |
| *Csf2* | Colony-stimulating factor 2 | 0.18 |
| *Tnf* | Tumor necrosis factor | 0.18 |
| *Bmp3* | Bone morphogenetic protein 3 | 0.24 |
| *Bmpr1a* | Bone morphogenetic protein receptor 1a | 0.26 |
| *Col11a1* | Collagen 11a1 | 0.27 |

**Supplementary Table 3:** Bone histomorphometric variables in 3-month-old WT and *Penk1^-/-^* mice

|  | **Males** | | | **Females** | | |
| --- | --- | --- | --- | --- | --- | --- |
|  | **WT** | ***Penk1^-/-^*** | **p value** | **WT** | ***Penk1^-/-^*** | **p value** |
| Oc.S/BS, % | 11.69 | 11.62 | 0.96 | 12.56 | 11.53 | 0.60 |
| Oc.N/BS (mm^-1^) | 3.52 | 3.38 | 0.74 | 4.96 | 4.96 | 0.99 |
| Ob.S/BS, % | 9.63 | 11.47 | 0.39 | 9.22 | 8.39 | 0.55 |
| Ob.N/BS (mm^-1^) | 6.03 | 6.68 | 0.52 | 5.37 | 5.29 | 0.71 |
| BFR(μm^2^/μm/day) | 1.11 | 1.24 | 0.54 | 1.19 | 1.17 | 0.95 |
| MAR (μm/day) | 2.52 | 2.92 | 0.20 | 3.28 | 3.62 | 0.62 |

**Supplementary Table 4:** Bone histomorphometric variables in 12-month-old WT and *Penk1^-/-^* mice

|  | **Males** | | | **Females** | | |
| --- | --- | --- | --- | --- | --- | --- |
|  | **WT** | ***Penk1^-/-^*** | **p value** | **WT** | ***Penk1^-/-^*** | **p value** |
| Oc.S/BS, % | 4.92 | 5.64 | 0.67 | 6.26 | 5.88 | 0.65 |
| Oc.N/BS (mm^-1^) | 1.87 | 2.46 | 0.36 | 1.77 | 2.27 | 0.29 |
| Ob.S/BS, % | 5.43 | 4.792 | 0.52 | 5.09 | 4.62 | 0.34 |
| Ob.N/BS (mm^-1^) | 3.32 | 3.25 | 0.89 | 3.63 | 3.49 | 0.67 |
| BFR(μm^2^/μm/day) | 0.45 | 0.37 | 0.53 | 0.47 | 0.39 | 0.25 |
| MAR (μm/day) | 1.68 | 1.73 | 0.85 | 1.15 | 0.99 | 0.07 |
